# Deregulation of Regulatory T Cells in Acute-on-Chronic Liver Failure: A Rat Model

**DOI:** 10.1155/2017/1390458

**Published:** 2017-01-15

**Authors:** Shunlan Ni, Shanshan Li, Naibin Yang, Xinyue Tang, Shengguo Zhang, Danping Hu, Mingqin Lu

**Affiliations:** ^1^Department of Infectious Diseases, Jinhua Municipal Central Hospital, Jinhua, Zhejiang, China; ^2^Department of Infectious Diseases, The First Affiliated Hospital of Wenzhou Medical University, Wenzhou Key Laboratory of Hepatology, Hepatology Institute of Wenzhou Medical University, Wenzhou, Zhejiang, China; ^3^Department of Infectious Diseases, Ruian City People's Hospital, The Third Affiliated Hospital of Wenzhou Medical University, Wenzhou, Zhejiang, China

## Abstract

*Aims*. Acute-on-chronic liver failure (ACLF) and acute liver failure (ALF) are similar in many respects during their acute exacerbation; however, ACLF generally has a poorer prognosis. We aimed to investigate the role and dynamic changes of regulatory T cell (Treg) and T helper 17 (Th17) cell proportions during ACLF progress.* Methods*. All rats were classified into two groups randomly: ACLF group and ALF group (control group). The rat model of ACLF was preestablished by intraperitoneal injection of carbon tetrachloride for 2 months. Then acute liver injury was induced by combined D-galactosamine and lipopolysaccharide. Six time points were examined before or after acute induction. Liver samples were performed with hematoxylin-eosin and Masson staining; circulatory Treg and Th17 cell frequencies were determined using flow cytometry assays; serum levels of alanine aminotransferase, aspartate aminotransferase, interleukin-10 (IL-10), and interferon-*γ* (IFN-*γ*) were examined.* Results*. In group ACLF, both Th17 cell proportion and IFN-*γ* level presented upgrade firstly and then descend latter tendency; the trends of Treg cell proportion and IL-10 level were observed to gradually decrease and became stable.* Conclusion*. The Treg cells played an important role in the immunologic mechanism during the process of ACLF. And the function of Treg cells in ACLF was defective.

## 1. Introduction

Liver failure is a serious condition that consists of jaundice, coagulopathy, encephalopathy, and metabolic derangement. Acute liver failure (ALF) develops in the absence of any preexisting liver injury, and acute-on-chronic liver failure (ACLF) develops with preexisting known or unknown chronic liver disease [1, 2]. ALF and ACLF show similar symptoms and signs which are difficult to distinguish each other during the acute phases. However, the prognosis is always poorer and mortality is higher in ACLF than in ALF [3]. Early-to-mid mortality of ACLF was found to be as high as 50%–90% [4, 5]. Recently, numerous clinical studies showed that immune response played an important role in the occurrence and development of ACLF [6–8]. Wasmuth et al. demonstrated that ACLF shows a similar immune paralysis as in severe sepsis [9]. However, the accurate pathogenesis and mechanisms remain to be elucidated.

As to immunity, the regulatory T cells (Treg cells) and IL-17-producing helper T cells (Th17 cells) received increasing concern in recent years [10–12]. Many studies demonstrated that Treg and Th17 cells were tightly associated with many human diseases, including the ACLF [13, 14]. The primary function of Treg cells is immune suppression. Deficiency or disruption of Treg cells was reported to result in autoimmune and inflammatory diseases in both humans and animals [15, 16]. Previous studies demonstrated that Treg cells can accumulate and expand at infection site where they exert immune suppression activity [17, 18]. Treg and Th17 cells share the same naive T cells and similar signaling pathways of cell differentiation. TGF-*β*, IL-6, IL-21, IL-23, INF-*γ*, and other cytokines play positive roles in promoting the process of Th17 cells differentiation and their function. Th17 cells protect against extracellular pathogens and induce tissue inflammation in host [19]. In addition, IFN-*γ* is a proinflammatory cytokine associated with accelerating hepatic inflammation and aggravating liver parenchymal damage [20].

The Treg and Th17 cells participate in the progression of hepatic failure. Niu et al. found that both circulating Treg and Th17 cell frequencies increased in hepatitis B virus- (HBV-) ACLF patients compared to the healthy ones [7]. However, others showed that the circulating Th17 cell proportion increased significantly and Treg cell changed without statistical significance [8, 21]. An interesting study demonstrated that the peripheral Th17 cell proportion increased in both acute hepatitis B virus (AHB) and HBV-ACLF patients compared with healthy controls (HC), and the Treg cell proportion significantly increased in AHB while it decreased with no statistical significance in HBV-ACLF compared with HC [6].

Combined, the clinical data of Treg and Th17 cells in ACLF are not consistent, and related experiment researches are absent. Therefore, we established an ACLF rat model and examined the frequencies of circulating Treg and Th17 cell at different time points after acute induction to investigate the dynamic changes of the two immunoregulation cells during the process of ACLF.

## 2. Materials and Methods

### 2.1. Animals

Male Sprague-Dawley rats weighing 160–180 g were purchased from Shanghai Laboratory Animal Center (Shanghai, China) and maintained in a 12 h light/dark cycle room and freely got food and water in animal centre of Wenzhou Medical University. All experimental procedures were conducted under the guidelines of Ethics Committee of Animal Care and Usage of Wenzhou Medical University. The license of rats was SYXK (zhe) 2014-0006.

### 2.2. Experimental Protocols

The rats were classified into two groups randomly: ACLF group (*n* = 80) and ALF group (control, *n* = 60). In ACLF group, chronic liver disease was preestablished using CCl_4_ (Bo Di, Tianjin, China) dissolved in peanut oil (volume, 1 : 1) by intraperitoneal injection once every three days for two months. The dosage regimen is 1.5 mL/Kg weight in the 1st month and 2.0 mL/Kg weight in the 2nd month. No treatment was received in ALF group. After two months, nine rats were dead in ACLF group during the chronic process while all rats were alive in control group. Then, six rats in each group were selected as the baseline control and named “0 h” subgroup. Next, the rest rats were treated with 500 mg/Kg weight D-GalN (Sigma-Aldrich, St. Louis, MO, USA) and 80 *μ*g/Kg weight LPS (Sigma-Aldrich) by intraperitoneal injection to induce acute liver failure.

**Figure 1:**
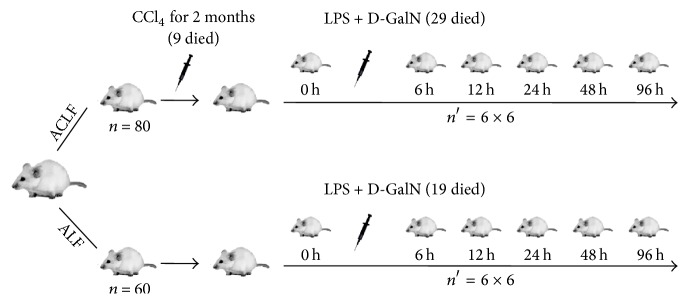
The process of making models of ACLF and ALF. All rats were classified into two groups randomly: ALF group and ACLF group. (1) ACLF group: the rats were treated with CCl_4_ dissolved in peanut oil (volume, 1 : 1) 1.5 mL/Kg weight in the 1st month and 2.0 mL/Kg weight in the 2nd month once every three days; six rats were selected as the baseline control and named “0 h”; the rest rats were treated with 500 mg/Kg weight D-GalN and 80 *μ*g/Kg weight LPS, and five time points were selected. (2) ALF group: no treatment was received for two months; subsequent treatments of ALF group were the same as ACLF group (ALF: acute hepatic failure; ACLF: acute-on-chronic liver failure; CCl_4_: carbon tetrachloride; D-GalN: D-galactosamine; LPS: lipopolysaccharide).

To examine the dynamic changes of immunity, five time points after injection were selected: 6 h, 12 h, 24 h, 48 h, and 96 h. In each time point, six rats in each group were randomly selected from the remaining live rat, respectively (*n*′ = 6). Then the rats were sacrificed for subsequent experiments. Approximately 1.5 mL blood sample was collected from caudal vein and liver tissues were isolated for later analysis. The serum was collected from the fresh blood after centrifugation at 3000 rev/min for 10 min. Of note, there were 29 rats in ACLF group and 19 rats in ALF group that died from liver failure in the first 96 hours after acute induction. The flow chart of operation procedures is showed in Figure 1.

### 2.3. Histology

Liver tissues were fixed in 4% paraformaldehyde (Solarbio, Beijing, China) for 48 h, embedded with paraffin, and cut into 4 *μ*m thick sections. Then, the slides were treated with hematoxylin and eosin (H&E) or Masson staining according to the manufacturer's protocols (Solarbio). Pathological changes were observed under an optical microscope.

### 2.4. Determination of Laboratory Data and ELISA

The serum levels of aspartate aminotransferase (AST) and alanine aminotransferase (ALT) were measured by Backman Kurt au5800 automatic biochemical analyzer (Beckman Kurt Co. Ltd., USA). Quantitative analysis of interleukin-10 (IL-10) and interferon-gamma (IFN-*γ*) levels in serum was performed using enzyme-linked immune sorbent assay (ELISA) kits according to the manufacturer's instructions (RB, USA).

### 2.5. Flow Cytometry

The peripheral blood mononuclear cells (PBMC) were isolated from whole blood using the lymphocyte isolation Kit (Hao yang BM, Tianjin, China), and 2 × 10^6^ cells were aliquoted to each tube. For Th17 cells analysis, cells were incubated in RPMI 1640 medium containing 10% FBS (Gibco, USA), 50 ng/mL phorbol 12-myristate 13-acetate (PMA), 1 *μ*g/mL ionomycin, 3 *μ*g/mL brefeldin A (BFA), and 1.4 *μ*g/mL monensin (Multi-Sciences, China) at 37°C with 5% CO_2_ for 6 hours. Cells were firstly stained with anti-CD4-FITC. Next, the cells were permeabilized using the Fix/Perm solution (eBioscience, USA) and stained with anti-IL-17-PE after fixation. For analysis of Treg cells, cells were firstly stained with anti-CD4-FITC and anti-CD25-APC synchronously and then stained with anti-Foxp3-PE after fixation and permeabilization as described above. All the antibodies used above were purchased from eBioscience (USA) and incubated for 30 min at 4°C in the dark. Finally, cells were resuspended in 1% paraformaldehyde and analyzed using a FACSCalibur (BD, USA) within 1 hour. Appropriate isotype controls and single staining controls were used. The data were analyzed using FlowJo7.6.1 software.

### 2.6. Statistical Analysis

Statistical analyses were performed using IBM SPSS Statistic v21.0 (IBM Co., Armonk, NY, USA). To show the relative changes of cell proportions and associated factors after acute insult, the normalized value relative to “0 h” was approved and calculated as follows: normalized  value  (%) = (time  point − 0 h)/0 h. Data are shown as mean ± standard deviation and compared using the independent *t*-test and analysis of variance (ANOVA). Value of *p* < 0.05 was considered to be a significant difference.

## 3. Results

### 3.1. Liver Histopathology

To examine the validity of ACLF models and observe the histopathological changes in acute phase after induction, liver tissues were examined with hematoxylin-eosin (HE) and Masson (M) staining, and the ALF models were used as controls.

**Figure 2:**
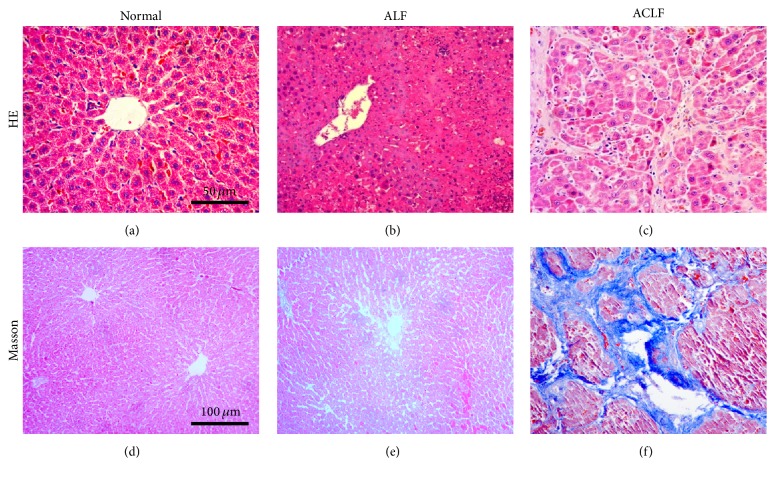
Pathological features of liver tissue under light microscope by HE (magnification 400x) and Masson staining (magnification 200x). Liver sections of normal, ALF, and ACLF group were stained with HE (a, b, and c) and Masson (d, e, and f), respectively. (a, d) Normal: the rats were from 0 h subgroup in ALF model; (b, e) “12 h” subgroup in ALF group; (c, f) “12 h” subgroup in ACLF group (HE: hematoxylin and eosin staining; ALF: acute hepatic failure; ACLF: acute-on-chronic liver failure).

We observed hard texture and nodular appearance of the ACLF livers. The slices showed cell swelling, necrosis, inflammatory cells infiltration, double-nucleus liver cells increasing, and multiple pseudolobule formation (Figures 2(c) and 2(f) show the tissues of “12 h” subgroup in ACLF group). The surfaces of livers in normal rats (from 0 h subgroup in ALF model) were smooth; hepatic cells were integral and hepatic lobules structures were clear in the specimens; fibroplasia was not observed under light microscope (Figures 2(a) and 2(d)). The liver tissue sections in group ALF showed necrosis and inflammatory cells infiltrating in portal duct areas without fibroplasia (Figures 2(b) and 2(e) showed the tissues of “12 h” subgroup in ALF group).

### 3.2. Laboratory Data

**Figure 3:**
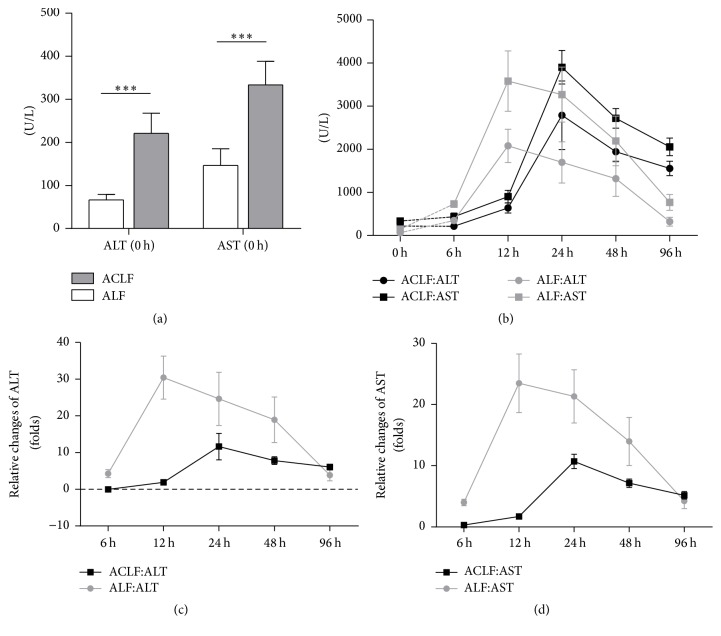
Serum ALT and AST levels. (a) The levels of ALT and AST at 0-hour point in ACLF group and ALF group; results were shown as means ± SD, ^*∗∗∗*^ *p* < 0.001. (b) The dynamic changes of ALT and AST in ACLF group and ALF group. (c, d) The relative changes of ALT and AST levels (ALT: alanine aminotransferase; AST: aspartate aminotransferase).

As serum biochemical changes reflect liver function, levels of ALT and AST were determined. The results are shown in Figure 3. ALT and AST levels significantly increased in ACLF group after CCl_4_ administration for 2 months compared with the control group at 0-hour time point (220.8 ± 47.0 versus 66.2 ± 12.8, *p* < 0.001; 333.3 ± 55.2 versus 146.3 ± 38.7, *p* < 0.001) (Figure 3(a)). The change trends of ALT and AST level in ACLF group were similar to the control group, which both increased firstly and began to decrease later. The levels of ALT and AST in ACLF group reached the peaks at 24-hour point, while the time advanced to 12-hour point in control group (Figure 3(b)).

To clearly observe the differences of changes in serum levels of ALT and AST between ACLF group and control group, we adjusted data by “0 h” subgroup, respectively. We found that the adjusted curves in group ACLF were gentler than the control group, which indicated that the ACLF group had high levels of ALT and AST at beginning and ending point of our observation (Figures 3(c) and 3(d)).

### 3.3. Peripheral Blood Treg and Th17 Cells

**Figure 4:**
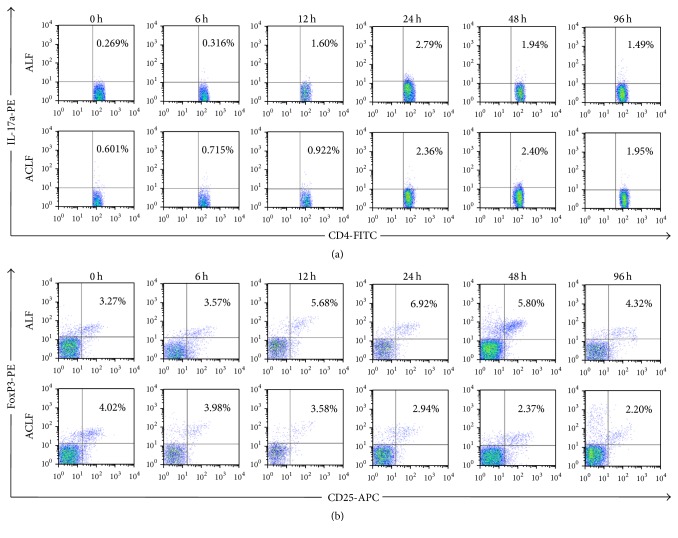
Th17 and Treg cells in peripheral blood in ACLF group and ALF group. (a) Th17 cells; (b) Treg cells (Th17 cells: T helper 17 cells; Treg cells: T regulatory cells).

Dynamic changes in Th17 and Treg cell proportions in peripheral blood were examined using flow cytometry assays at consecutive time points (Figures 4(a) and 4(b)).

**Figure 5:**
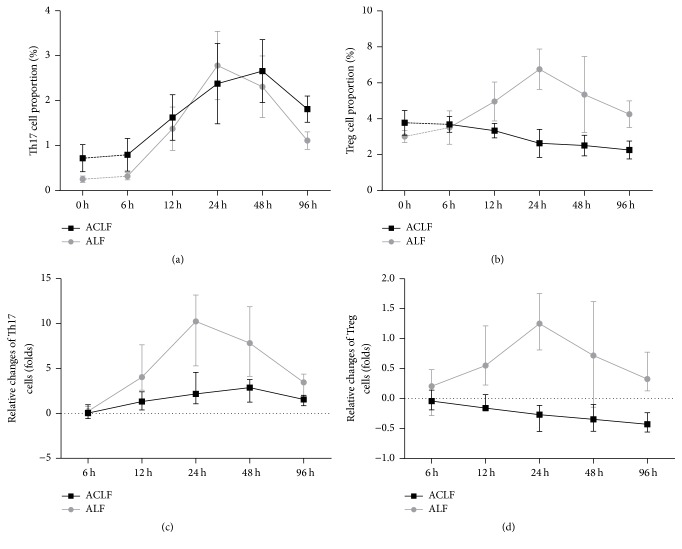
The changes of Th17 and Treg cell proportions. (a, b) Th17 and Treg cell proportions; results were shown as means ± SD. (c, d) Relative changes of Th17 and Treg cells.

Firstly, both Th17 (0.72 ± 0.30% versus 0.25 ± 0.07%, *p* = 0.012) and Treg cell (3.78 ± 0.69% versus 3.01 ± 0.33%, *p* = 0.043) frequencies in ACLF group were significantly higher than the control group before acute induction (at 0-hour point). Then, the corresponding changing curves were analyzed. In Th17 cells, the curves of the two groups were intertwined with each other. The curve of ACLF group elevated significantly and reached a peak at 48-hour point, while the peak was advanced to 24-hour point in control group. The proportion in “96 h” subgroup was lower than in “48 h” subgroup of ACLF group (1.81 ± 0.29% versus 2.66 ± 0.70%, *p* = 0.030) (Figure 5(a)).

In Treg cells, different trends were observed in the two groups. The cell proportion in rats with ALF increased firstly and began to decrease after 24 hours, while the trend in rats with ACLF was decreasing at the very beginning. Over time, the proportion of Treg cells in ACLF group decreased significantly first and finally reached a steady state (Figure 5(b)).

To observe the change trends of Treg and Th17 cell frequencies more clearly, we adjusted different data by “0 h” subgroup, respectively (Figures 5(c) and 5(d)). The more gently the curves were, the smaller the ranges of the curves changed. We found that the adjusted curves of Treg and Th17 cell proportion in ACLF group were gentler than the control group. Our data showed that the maximal proportion of circulatory Th17 cell increased about 2.88-fold in ACLF group and that in the control group increased about 10.25-fold, respectively (Figure 5(c)).

**Figure 6:**
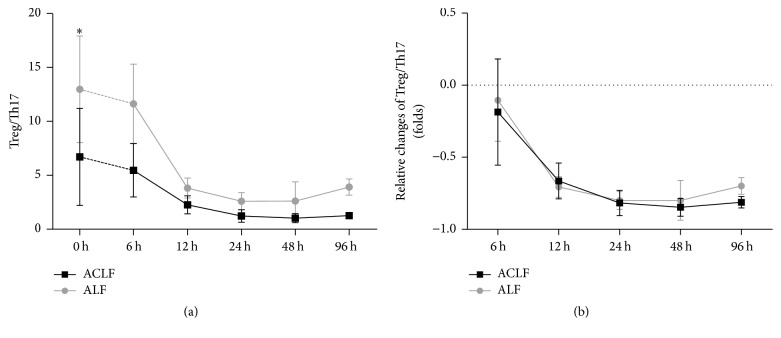
Change of Treg/Th17 ratio. (a) Treg/Th17 ratio in ACLF group and ALF group. (b) Relative changes of the ratios; ^*∗*^ *p* < 0.05.

The balance between Treg and Th17 cells could reflect the regulating function of immune system. In recent study, we examined changes in Treg to Th17 cells ratio (Figures 6(a) and 6(b)). Our data showed that the ratio in ACLF group decreased significantly compared with the control group at 0-hour point (6.71 ± 4.50 versus 12.97 ± 4.93, *p* = 0.045). The changes of Treg/Th17 ratio both declined in the two groups (Figure 6(a)). These data indicated that the balance of Treg and Th17 cells was disrupted in ACLF group and also in control group. And we found the ratio tended to be stable after 12 hours in ACLF group, while the ratio had an increasing trend after 48 hours in control group (*p* > 0.05).  Figure 6(b) shows the relative changes of Treg/Th17 ratio.

### 3.4. Immunologic Cytokines

**Figure 7:**
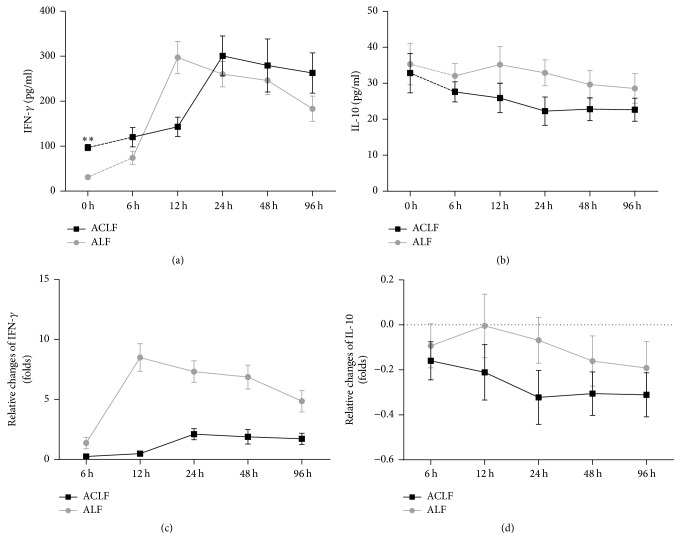
Serum levels of IFN-*γ* and IL-10. (a, b) Changes of IFN-*γ* and IL-10 level in serum in ACLF group and ALF group. (b) IFN-*γ* and IL-10 levels were normalized by “0 h” subgroup, respectively; ^*∗∗*^ *p* < 0.01. (IFN-*γ*: interferon-*γ*; IL-10: interleukin-10).

IFN-*γ* and IL-10 played a role in differentiation of Th17 and Treg cells. Here, serum levels of IFN-*γ* and IL-10 were measured by ELISA kit and data were summarized in Figure 7. Our data showed the level of plasma IFN-*γ* increased while plasma IL-10 level had no significant decrease in ACLF group compared to control group at 0-hour point (96.87 ± 6.84 versus 31.29 ± 3.28, *p* < 0.001; 32.84 ± 5.48 versus 35.33 ± 5.75, *p* = 0.459).

Our results also showed plasma IFN-*γ* level significantly increased first (*p* < 0.05) and reached a peak at 24-hour point and then decreased with no significance (*p* > 0.05) in ACLF group. Meanwhile, plasma IFN-*γ* level in control group had a similar trend with ACLF group, but it decreased significantly (*p* < 0.05) with the peak advanced to 12-hour point (Figure 7(a)). There was an unconspicuous peak at 12-hour point and a subsequently significant decrease of plasma IL-10 level in control group, while plasma IL-10 level slowly decreased during ACLF progress and finally reached a steady state (Figure 7(b)).

The curves normalized by “0 h” data, respectively, showed less change of IFN-*γ* in ACLF group than control group (Figure 7(c)). We found relative amplitude of change in IL-10 level was clearer after the data adjusted, and IL-10 levels in ACLF group reduce more than control group (Figure 7(d)).

These results suggested that IFN-*γ* was associated with hepatic failure progress and still maintained a higher level in the later stage of ACLF. Our data also indicated that gradual decreasing of IL-10 level plays a role in immunosuppression.

## 4. Discussion

Clinically, acute-on-chronic liver failure (ACLF) is a potentially lethal disease with a higher mortality than acute liver failure (ALF), characterized by the rapid deterioration of liver function [3, 22]. Recently, much attention has been focused on the dysfunction of immunity as a potential mechanism for the progression of ACLF, especially the roles of Treg and Th17 cells which play opposite roles in immunoregulation [21, 23]. Imbalance between the two types of CD4+ T cells which associated with immune system disorders is of vital importance in the pathogenesis of ACLF [6, 7]. In the current study, we investigated the dynamic changes of circulating Treg and Th17 cells and associated factors during acute liver failure progression in a rat model of ACLF with ALF model as control.

Previous publications demonstrated that Th17 cells stimulate production of numerous inflammatory chemokines and proinflammatory cytokines and promote recruitment of granulocytes in tissues [24]. And Ivanov et al. showed that Th17 cells participate in the development of inflammation and autoimmune diseases [25]. The Th17 subset was also reported to play an important role in many liver diseases, including chronic hepatic failure, autoimmune liver disease, and HBV or HCV-associated liver disease in both patients and animals [26–30]. In this study, rats in 0 h subgroup of ACLF group showed a significantly higher level of the circulating Th17 cells compared to the control, which was supported by many previous studies [7, 8, 28]. Then the dynamic changes of Th17 cells were examined after acute insult. The Th17 cell proportion increased first and then decreased with a peak at about 36 h in both the ACLF and ALF group, of which the curves are close to each other. However, the ACLF group demonstrated less intensive relative changes than the ALF group when adjusted to the baseline value.

Besides, the variation trend of IFN-*γ* level showed a similarity with the trend of Th17 cells. The Th17 cells and IFN-*γ* levels showed less variation range in ACLF group compared to that in control group. Previous studies had demonstrated that high level of IFN-*γ* is involved in severe liver damage in mice and in human with ACLF [31, 32], which accorded with our observations. These results suggested that Th17 cells and IFN-*γ* participated in promoting and maintaining inflammatory response and aggravated the liver damage.

As mentioned above, the primary function of Treg cells is to negatively regulate the immunoreactions, which play an important role in maintaining the balance of the immune system [16, 33]. Evidence showed that Treg cells in spleen decreased along with the aggravation of liver fibrosis in mice [28], while the circulating Treg cells significantly increased in CHB patients [34]. Similar to Th17 cells, our data showed that the circulating Treg cells in CLF rats were higher than the normal ones. Of note, Treg cells in ACLF group keep decreasing after acute induction, while they increased in the first 24 hours and then began to decrease in ALF group.

The changes of Treg levels in ACLF patients are controversial. Several studies had characterized peripheral Treg cell proportion significantly higher in ACLF patients than in CHB patients [7, 23, 35]. Conversely, other studies found peripheral Treg cells decreased in ACLF patients from CHB [6, 8, 21]. Our data suggested that Treg cell proportion remained decreasing and then reached a stable state during the progress of ACLF, while the proportion increased first and then decreased in control group. We also found that the concentration of Treg-associated cytokine (IL-10) decreased like the dynamic change of Treg cell proportion in ACLF group. The increase of Treg cell proportion and Treg-associated cytokines could inhibit T cell responses, which play an indispensable role in regulating inflammation of liver tissue and controlling liver pathology [36]. Thus, we speculated that decreasing Th17 cell proportion after 24 hours and downregulating Th17-associated cytokine after 12 hour in control group which was different from the changes in ACLF group were associated with the elevation of Treg cell proportion and upregulation of IL-10 level in control group. The Treg cell proportion in ACLF group was supposed to increase to balance the increasing Th17 cell proportion as in control group, but it was downregulated. Therefore, we speculated that the function of Treg cell in an ACLF model was defective.

These results gave a clue that Treg cell may be in a key position in immunologic mechanism of hepatic failure. First, the increase of Treg cells may be a feedback to the elevation of Th17 cells and proinflammatory factors, and its function is to suppress the immune response finally making a new balance of Th17/Treg axis in chronic course of liver failure. Second, proinflammatory immunity was markedly upregulated while anti-inflammatory immunity downregulated during ACLF progress. Thus, we speculated the immunoregulation function of Treg cells was damaged. Third, in later stages of ACLF, the changing trends of Th17 cell proportion and proinflammatory cytokines level were both descending. We indicated that the low-level state of Th17 cells proportion and proinflammatory cytokines in later stage of ACLF was immune paralysis. Wasmuth and coworkers demonstrated that patients with ACLF have immune paralysis like the patients with sepsis [9]. Some researchers showed that immune paralysis had a largely reduced capacity to produce anti-inflammatory cytokines after stimulation by LPS [37].

In conclusion, we indicated that the proportion of circulating Treg cells did not increase but decreased while Th17 cells increased in the progress of ACLF. The function of Treg cells in ACLF may be defective after a chronic process of liver failure. However, there were some limitations in this study. Firstly, our conclusions are based on the analysis of the typical model of drug-induced liver failure, and the results based on virus-induced or other kinds of liver failure remain to be explored. Secondly, a single dosage of drug and limited time points were applied. More information might be revealed under different drug concentrations for acute insult or using more intensive checkpoints. In addition, Treg and Th17 cell proportions were detected from peripheral blood, and in situ testing technique will be approved in our further studies.
